# SEEG‐RFTC in patients with refractory focal epilepsy: real‐world outcomes from 121 cases

**DOI:** 10.1002/acn3.52117

**Published:** 2024-07-05

**Authors:** Qiangqiang Liu, Yanqing Cai, Ziyu Mao, Wenze Chen, Bin Chen, Wenzhen Chen, Chencheng Zhang, Yong Lu, Jiwen Xu, Dake He

**Affiliations:** ^1^ Clinical Neuroscience Center, Ruijin Hospital Luwan Branch Shanghai Jiao Tong University School of Medicine Shanghai P. R. China; ^2^ Clinical Neuroscience Center Comprehensive Epilepsy Unit, Department of Neurosurgery, Ruijin Hospital Shanghai Jiao Tong University School of Medicine Shanghai P. R. China; ^3^ Department of Children Neurology Xinhua Hospital Affiliated to Shanghai Jiao Tong University School of Medicine Shanghai P. R. China

## Abstract

**Objective:**

Radiofrequency thermocoagulation (RFTC) has emerged as an effective and safe treatment method for patients with refractory focal epilepsy, when stereo‐electroencephalography (SEEG) is implanted. Although real‐world research results are still limited, a considerable number of patients have shown favorable outcomes with this less invasive method. This study aims to describe the outcomes and predictive factors of SEEG‐RFTC in real‐world research.

**Methods:**

A retrospective observational study was conducted on patients in the authors' epilepsy center. In total, 121 patients who underwent RFTC were included in the study. Post‐RFTC outcomes were evaluated using the seizure‐free rate and response rate (seizure frequency reduction more than 50%). Predictive factors influencing post‐RFTC outcome were considered by comparing different variables.

**Results:**

The mean follow‐up period was 18.3 months. Eighty‐two patients (67.8%) were responders and 54 (44.6%) were seizure free. In 36 patients with malformation of cortical development, the seizure‐free rate and the response rate were 69.44% and 83.33%, respectively. In 20 patients with hippocampal sclerosis, 19 patients were responders and 14 (70%) patients were seizure free at the last follow‐up. The MRI feature and etiology of epilepsy are correlated with the outcome. MR‐positive is a predictive factor for seizure freedom (*p* < 0.01) and responders (*p* < 0.01). Other factors have no predictive value for post‐RFTC outcome.

**Interpretation:**

SEEG‐RFTC is a safe procedure and yields favorable outcomes in numerous cases of focal DRE. The MRI feature and etiology of epilepsy are correlated with the seizure‐free rate and response rate. And MRI positivity is the predictor for good RFTC outcome.

## Introduction

Epilepsy is one of the most frequent neurological diseases, affecting nearly 1% of the population,^1^ and approximately 30%–40% of the worldwide epilepsy patients show resistance to anti‐seizure medication (ASM) and are diagnosed with drug‐resistant epilepsy (DRE).^2^ Patients with focal DRE may be eligible for epilepsy resection and reach seizure control in selected individuals.^2^, ^3^ Nevertheless, surgery could be prohibited because of the overlap of the resected area with functional areas or extended networks. Moreover, resection surgery is highly invasive, and cognitive impairment, psychiatric disorders, and cognitive deficits are common complications that may reduce patients' quality of life after surgery.^4^

Stereo‐electroencephalography (SEEG) may be crucial for assessing the feasibility of resecting the seizure onset zone (SOZ), particularly when there are challenges in locating the SOZ accurately.^5^ In conjunction with the SEEG evaluation, the presence of multiple electrode contacts within the seizure onset zone offers the potential for thermocoagulation without the need for additional invasive procedure. SEEG‐guided radiofrequency thermocoagulation (RFTC), was first described by Guenot et al. in 2004,^6^ has emerged in the last few decades as an alternative therapy to conventional resection.^6^, ^7^ According to a recent meta‐analysis including accounts for 233 patients with focal cortical dysplasia (FCD), about 90% of subjects show more than 50% decrease in seizure frequency after RF‐TC, and 52% of them achieve seizure freedom.^8^ To date, SEEG‐guided RFTC has also been applied in patients with focal lesions such as mesial temporal lobe epilepsy,^9^, ^10^, ^11^, ^12^ insular epilepsy,^13^, ^14^ periventricular nodular heterotopias(PNH),^15^, ^16^ and hypothalamic harmatomas.^17^, ^18^

Previous studies have focused on the clinical outcome of a single etiology. In this study, we aimed to describe the outcomes of SEEG‐guided RFTC in a series of patients with different etiologies from real‐world research, seeking to better define the indications of the procedure and possible factors associated with a favorable response.

## Materials and Methods

### Patients selection

We conducted a retrospective observational investigation at Ruijin Hospital in China, focusing on patients who underwent SEEG‐guided RFTC for refractory focal epilepsy between June 2016 and June 2023. The study was conducted at a single center. The inclusion criteria for the study were as follows: (1) confirmed diagnosis of drug‐resistant focal epilepsy, and (2) a minimum postoperative follow‐up duration of 5 months.

One hundred and twenty‐seven epilepsy patients underwent SEEG implantation surgery, 121 of which underwent radiofrequency therapy and were included in this study. We retrospectively reviewed the medical history, magnetic resonance imaging (MRI), and ^18^F‐fludeoxyglucose (^18^F‐FDG) positron emission tomography/magnetic resonance imaging (PET/MRI) data, video‐EEG, and SEEG recordings of all included patients to collect information on demographic data, epilepsy history, procedures of SEEG and RFTC, as well as post‐RFTC Engel Class and response (seizure frequency reduction more than 50%).

The Ethics Committee of Ruijin Hospital Luwan Branch reviewed and approved this study (No. LWEC2023040). All participants provided informed consent to participate in this study.

### SEEG electrodes implantation and seizure investigation

For all patients undergoing SEEG assessment, the presurgical investigation included at least long‐term video‐EEG recording, high‐resolution 3.0 T MRI with an epilepsy protocol, ^18^F‐PET/MRI, and neuropsychological evaluation. All cases were discussed by the multidisciplinary team of specialists in epilepsy, and all patients were selected for evaluation by SEEG, when they experienced focal drug‐resistant epilepsy and noninvasive investigations failed to adequately localize the epileptogenic zone (EZ). Based on the results of the pre‐surgical evaluation, a hypothesis about the EZ was raised and target SEEG planning was conducted, as previously described.^19^ For patients with suspected medial temporal lobe epilepsy (MTLE), three electrodes were inserted orthogonally in the direction of amygdala, and ventral head and anterior body of hippocampus. If sclerosis or atrophy was found in the dorsal hippocampus, an electrode would also be implanted.

Intracerebral electrodes were stereotactically implanted using robotic guidance (Sinovation, Beijing, China, uNav‐Brian 550, United, China or ROSA, Zimmer Biomet, Warsaw, IN, USA). Electrode contacts (HKHS, Beijing, China) had the following characteristics: 8–18 contacts, 0.8 mm in diameter, 2 mm long, and 1.5 mm apart. After electrode implantation, the patients underwent intraoperative head computed tomography (CT) scan to confirm that the electrodes implantation position had met the requirements.^20^ The anatomical location of the electrode contacts was also identified through fused the intra‐operative CT to the preoperative MRI in 3D Slicer (version 4.10.2) software.^21^

All patients underwent the intracranial recording (14–28 days) using an intensive 256 channels video‐EEG monitoring system (EEG‐1200C, Nihon Kohden, Japan) on the second day after surgery. To shorten the duration of recording, patients could reduce or withdraw ASMs to induce spontaneous seizures.

Once an adequate number of spontaneous seizures were captured, cortical electrical stimulation would be performed. The mapping was performed every two contiguous contacts by constant electric stimulator (MS‐120BK, Nihon Kohden, Japan). The parameters were as follows: 50HZ, 3 s, biphasic square‐wave current progressively raised from 0.1 to 5.0 mA. The electrically elicited eloquent areas and seizures would be used for diagnosis.

### SEEG‐guided RFTC procedure

The SEEG‐guided RFTC procedure was performed at the end of the recording before electrodes' withdrawal. RFTC was offered to most patients undergoing SEEG assessment and routinely to every patient since June 2016 in our center. Indications for RFTC was that the hypothetical SOZ had been confirmed by SEEG and resection plan had been conducted combining MRI and PET data. The discharge area before the appearance of semiology would be considered as a rapid propagation area and classified as a resection area (at a distance closer to the SOZ, it does not cause significant functional defect). All contacts located in the resection area were considered to be the targets of coagulation. For cases of MTLE, the resection plan was the standard anterior temporal lobe resection. All electrode contacts within the resection area would receive coagulation, including all contacts in the hippocampus and amygdala. Especially for epilepsy caused by FCD, contacts located in the functional area (within the lesion) were also considered for coagulation under the guidance of diffusion tensor imaging and patient's consent. Most of these functional impairments were temporary and cloud be recovered.^22^

The effective distance between contacts for extending thermo‐lesions was found to be approximately 7 mm.^23^ Two contiguous contacts in one electrode and two contacts situated on adjacent electrodes were used to for coagulation. The procedure was performed using a radiofrequency lesion generator (R2000B, BNS, Beijing, China). The parameters were as follows: current power progressively raised from 3.5 to 7.0 W within 30–60 s. We continued SEEG monitoring for a few days after coagulation to observe changes in ictal and interictal activity. Finally, the SEEG electrodes were removed and the patient was discharged from the hospital within 24 h after head CT scan confirmed no complications occurred.

### Data collection and statistical analysis

The seizure frequency, Engel Class, complications, and subsequent surgery were evaluated through various means including telephonic inquiries as well as outpatient and inpatient examinations. The mean, standard deviation, and range were calculated for numeric variables, and the absolute and relative frequencies were computed for categorical variables. To assess differences in RFTC outcomes among the variables, the chi‐squared significance test (*χ* ^2^ test) was used to compare qualitative data, and an unpaired Student's *t*‐test was used to compare quantitative data. The binary logistic regression analysis was used to analyze the impact of characteristics on RFTC outcomes. The Kaplan–Meier curve was used to analyze the seizure‐free and response rates in different etiologies. All statistical analyses were performed using the SPSSAU project (2023) (version 23.0, https://www.spssau.com), and statistical significance was set at *p* < 0.05.

## Results

### Patient characteristics

A total of 121 focal DRE patients underwent the application of RFTC, including 54 men and 67 women, with a mean age of 23.00 ± 11.32 years (ranging from 4 to 58 years) and a mean duration of seizure disorder of 10.34 ± 7.88 years (ranging from 1 to 35 years). The seizure frequency varied from daily to monthly. Eighty‐five patients (70.25%) showed positive MR results, and the other 36 patients (29.75%) were MRI negative. All patients underwent PET/MR scans, with 5 cases showing negative PET results. Malformation of cortical development (MCD) (*n* = 36, 29.75%, include 24 patient with FCD, 12 with gray matter heterotopia, and 3 with tuberous sclerosis) and hippocampal sclerosis (HS) (*n* = 20, 16.53%) are the most common definite causes. Unilateral implantation of depth electrodes was performed in 93 patients (76.86%) (51 on the left and 42 on the right), and bilateral implantation in 28 patients (23.14%). The mean SEEG electrode number in the patients was 11.18 ± 2.24 (ranging from 4 to 17). The demographic data and clinical characteristics of 121 patients are shown in Table 1.

**Table 1:** Population characteristics.

Characteristics	Total patients
Number (*N*)	121
Male/female (*N*)	67/54
Age (years, mean ± SD)	23.00 ± 11.32
Duration of epilepsy (years, mean ± SD)	10.34 ± 7.88
Follow‐up period (months, mean ± SD)	18.67 ± 13.60
Seizure frequency	
Daily	39 (32.23%)
Weekly	36 (29.75%)
Monthly	46 (38.02%)
MRI feature	
Positive	85 (70.25%)
Negative	36 (29.75%)
PET/MRI feature	
Positive	116 (95.87%)
Negative	5 (4.13%)
Etiology of epilepsy	
Undefined	36 (29.75%)
Malformation of cortical development (MCD)	36 (29.75%)
Hippocampal sclerosis (HS)	20 (16.53%)
Brain injury (trauma or hemorrhage)	14 (11.57%)
Low‐grade tumors	11 (9.09%)
Encephalitis	2 (1.65%)
Hypoxic–ischemic encephalopathy	2 (1.65%)
SEEG implantation side	
Left side	51 (42.15%)
Right side	42 (34.71%)
Bilateral side	28 (23.14%)

### Seizure outcomes after RFTC

The mean follow‐up period after RFTC (and before ultimate resection or neuromodulation if it applies) was 18.67 ± 13.60 months (range 4–54 months). Thirty‐nine patients (32.23%) underwent subsequent resection or neuromodulation, and the follow‐up time were set to the time before surgery.

**Figure 1:**
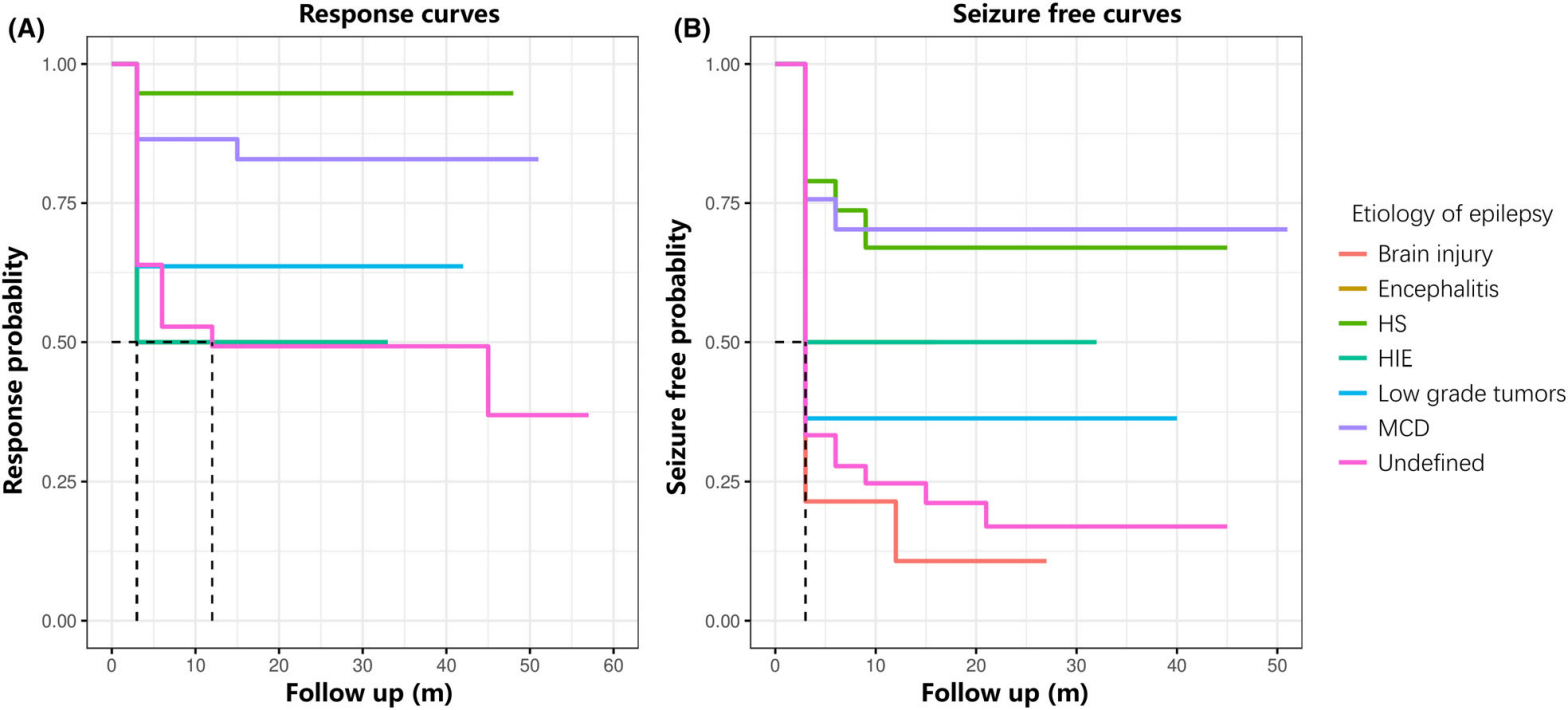
The response and seizure‐free curves of all patients. The Kaplan–Meier curves and probabilities were analyzed based on different etiologies. The response curves and probability (A) and the seizure‐free curves and probability (B) show that MCD and HS group have more better effects. Patients who undergo subsequent surgery will have their survival time calculated based on preoperative follow‐up time. HIE, hypoxic–ischemic encephalopathy; HS, hippocampal sclerosis; MCD, malformation of cortical development.

At last follow‐up, a total of 54 patients (44.63%) became seizure free after RFTC and were classified as Engel class I. Engel class II and III were achieved in 27 and 38 patients, respectively. Three patients who show no worthwhile improvement were classified as Engel class IV. Eighty‐two patients (67.77%) showed at least a 50% reduction in seizure frequency and identified as responders. In subgroup analysis, the response rate and seizure‐free rate of patients in the positive MRI group were higher than those in the negative group, with 76.47% versus 47.22% and 55.30% versus 19.44%, respectively. The Kaplan–Meier curves and probablities of response and seizure free based on different etiologies are shown in Fig. 1. The HS and MCD groups had more stable and better effects in terms of seizure‐free and response rate.

### Complications

The vast majority of patients reported experiencing discomfort throughout the RFTC procedure, manifesting as sensations of electromagnetic sound, heat, and pain associated with coagulation. In addition, there are also 6 patients experienced seizures during RFTC. Five patients who underwent RFTC in the precentral gyrus experienced temporary motor deficits in their contralateral limbs, which resolved spontaneously. Twelve patients experienced subclinical cerebral hemorrhage in the coagulation area found in CT images after electrodes removal. None of these patients required surgical intervention for the evacuation of the subclinical hematomas.

### Factors influencing RFTC outcome

There were significant differences in the MRI feature and etiology of epilepsy between patients that became responders and nonresponders, also between seizure freedom and not. The remaining characteristics of patient (gender, age, seizure frequency, duration of epilepsy, SEEG implantation side, electrode number, lobe, and side of SOZ) did not show statistically significant differences not only between responders and nonresponders, but also between seizure freedom and not (Tables 2 and 3). The binary logistic regression analysis showed that the MRI feature had a significant positive impact on response (*z* = 3.022, *p* < 0.01, odds ratio (OR) = 8.003) and on seizure freedom (*z* = 4.205, *p* < 0.01, OR = 16.079).

**Table 2:** Seizure freedom and responder at the last visit among patients with numeric variables.

Characteristics	Seizure freedom (mean ± SD)		*t*	*p*	Responder (mean ± SD)		*t*	*p*
	Yes (*n* = 54)	No (*n* = 67)			Yes (*n* = 82)	No (*n* = 39)		
Age (years)	22.07 ± 12.56	23.76 ± 10.24	0.797	0.428	22.54 ± 11.24	24.00 ± 11.55	0.663	0.508
Duration of epilepsy (years)	9.39 ± 7.69	11.10 ± 8.00	1.192	0.235	9.77 ± 7.97	11.54 ± 7.66	1.156	0.25
Electrode number	11.39 ± 2.29	11.01 ± 2.21	−0.911	0.364	11.16 ± 2.34	11.23 ± 2.05	0.165	0.869

**Table 3:** Seizure freedom and responder at the last visit among patients with categorical variables.

Characteristics	Sum	Seizure freedom		*χ* ^2^	*p*	Responder		*χ* ^2^	*p*
		Yes (54)	No (67)			Yes (82)	No (39)		
Gender									
Female	54	25	29	0.11	0.74	35	19	0.39	0.533
Male	67	29	38			47	20		
Seizure frequency									
Daily	39	17	22	0.14	0.933	26	13	0.071	0.965
Weekly	36	17	19			25	11		
Monthly	46	20	26			31	15		
SEEG implantation side									
Left	51	25	26	1.305	0.521	34	17	0.06	0.97
Right	42	19	23			29	13		
Bilateral	28	10	18			19	9		
Lobe of SOZ									
Temporal	53	22	31	4.021	0.403	36	17	3.436	0.488
Frontal	40	19	21			25	15		
Parietal	14	9	5			11	3		
Occipital	10	3	7			6	4		
Insular	4	1	3			4	0		
Side of SOZ									
Left	63	29	34	0.105	0.746	42	21	0.073	0.787
Right	58	25	33			40	18		
MRI feature									
Negative	36	7	29	13.153	0.000**	17	19	9.904	0.002**
Positive	85	47	38			65	20		
Etiology of epilepsy									
Undefined	36	7	29	28.998	0.000**	17	19	20.844	0.004**
MCD	36	25	11			30	6		
Hippocampal sclerosis	20	14	6			19	1		
Brain injury	14	2	12			7	7		
Low‐grade tumors	11	4	7			7	4		
Encephalitis	2	1	1			1	1		
HIE	2	1	1			1	1		
Sum	121	54	67			82	39		

HIE, hypoxic–ischemic encephalopathy; MCD, malformation of cortical development; SEEG, stereo‐electroencephalography; SOZ, seizure onset zone.

** *p* < 0.01.

## Discussion

As an additional treatment method following SEEG monitoring, RFTC has alleviated the pain of surgical resection for many patients. This is the real‐world study to focus on describing the clinical outcome of SEEG‐RFTC in focal DRE with multiple etiologies in Asia. A total of 54 patients (44.63%) became seizure free and 82 patients (67.77%) became responders after RFTC procedure, at the mean 18 months follow‐up.

Temporal lobe epilepsy, the most common type of epilepsy, is also the epilepsy with the highest utilization of RFTC techniques, especially for mesial temporal lobe epilepsy with hippocampal sclerosis (MTLE‐HS), which shows significant efficacy^9^, ^11^ and milder neuropsychological impacts.^12^ A study reported that 28 patients with mesial temporal lobe epilepsy with MTLE‐HS who underwent RFTC, the Engel Class I outcome rate was 72.41% at 1 year and 42.86% at 5 years.^11^ Our study also produced similar results: among 20 patients with MTLE‐HS, 14 (70%) were seizure free after a follow‐up of 19.3 months, and 19 (95%) were considered responders.

The responsiveness to RFTC is lower in patients with MRI‐negative focal DRE compared to those with positive. In our study, we included 36 patients with MRI‐negative focal DRE. Among these patients, 19.4% achieved seizure freedom, and 47.2% responded positively to the RFTC. These findings were consistent with previous studies.^7^, ^24^ Oliveira et al. reported the RFTC effects of 31 cases of MRI‐negative focal DRE with a mean follow‐up of 30.9 months.^24^ Among these patients, 25.8% were seizure free, and 45.2% were responders.

PET/MRI is a valuable tool for localizing epileptic lesions in patients who have negative MRI results.^25^ Among our 36 patients with negative MRI results, all showed positive results on PET/MR, which assisted us in formulating the SEEG implantation plan. A study reported the results of SEEG‐guided RFTC in 27 patients with negative MRI results using PET/MRI for epileptic lesion localization.^7^ Fifteen patients (55%) achieved a seizure‐free status at 2‐year follow‐up period. The findings of this study appear to be superior to our results. This might be because the MRI negativity they described was not based on the epilepsy protocol, while the protocol was used in the PET/MRI. There were actually 14 patients who were MRI‐positive.

**Figure 2:**
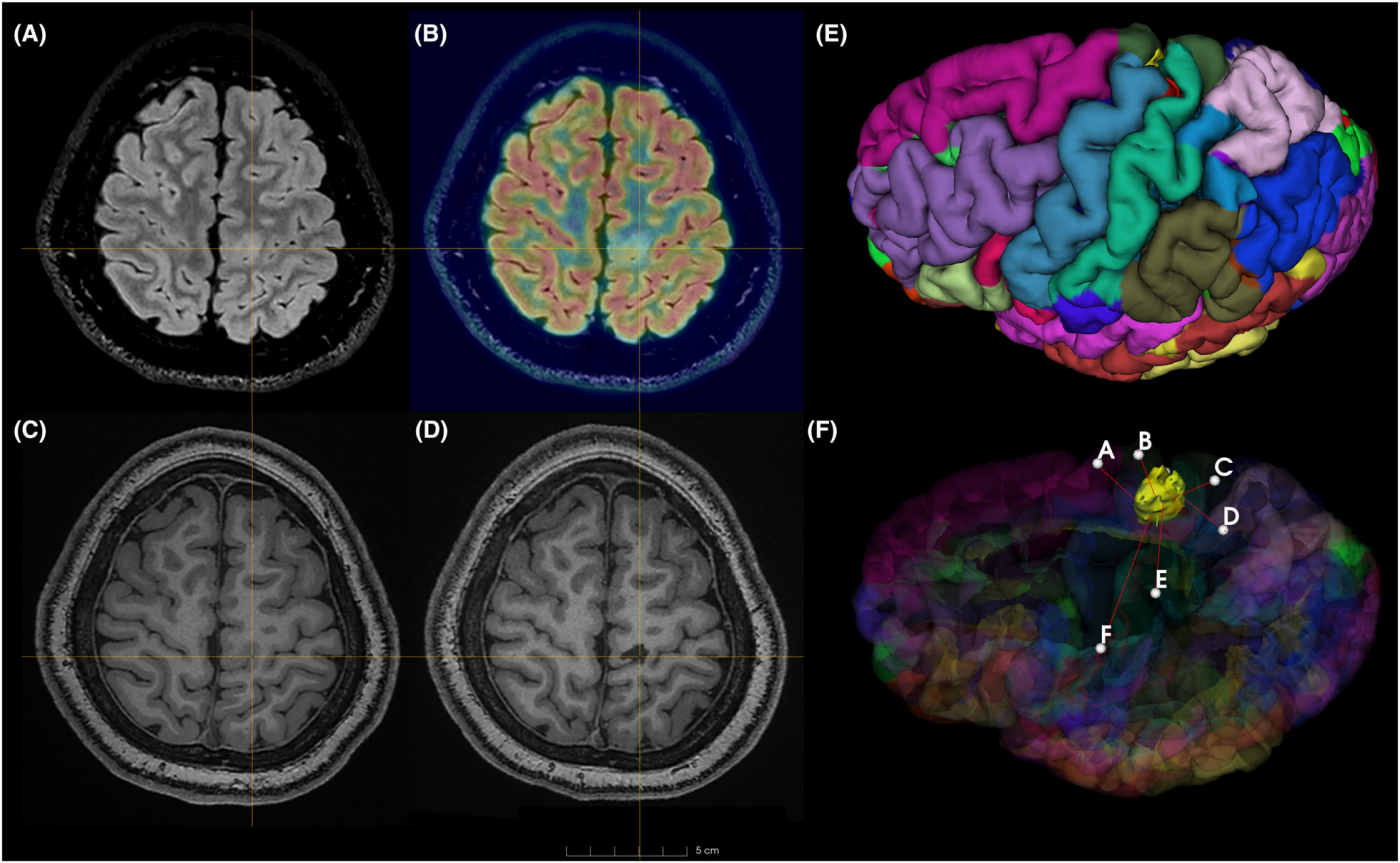
Preoperative and postoperative image evaluation (Patient 119). A child patient presented with daily seizures of the right limbs. On a Flair sequence (A), FCD in the left precentral gyrus was identified (cross point). PET (B) with Flair showed decreased metabolism in the same area. T1 sequence (C) revealed FCD lesioning. Following RFTC (D), T1 sequence showed complete FCD lesioning. All images were registered and displayed in the same plane. The three‐dimensional model of the left hemisphere (E), parcellation of the cortex marked by different colors. The FCD model (yellow) was overlaid with a transparent model of the hemisphere (F) and six SEEG models were placed to cover the FCD lesioning extensively. After RFTC, he remained seizure free at the last follow‐up visit (6 months) without any significant neurological deficit. FCD, focal cortical dysplasia, RFTC, radiofrequency thermocoagulation, SEEG, stereo‐electroencephalography.

MCD is another major etiology of epilepsy, including FCD, gray matter heterotopia, tuberous sclerosis, and periventricular nodular heterotopia.^26^ Guo et al. reported the outcomes of 22 patients with functional area FCD epilepsy after SEEG‐guided RFTC, with 17 (77.3%) patients achieving Engel Class I results.^27^ A meta‐analysis reported the outcome of FCD and PNH‐related DRE after SEEG guided RFTC.^28^ The seizure‐free rate and for 67 patients with FCD and 23 patients with PNH were 18% and 38%, respectively, while the response rate for FCD and PNH were 45% and 81%. In our study, there were 36 cases diagnosed with MCD. After receiving RFTC, the seizure‐free rate and the response rate were 69.44% and 83.33%, respectively. In our subgroup analysis, the seizure‐free rate and response rate for 21 cases of FCD were 66.7% and 90.48%, respectively, and the seizure‐free rate and response rate for 11 cases of heterotopia were both 75%. This might be related to the number and method of electrodes we implanted. For these focal lesions that were MRI‐positive, we employed a more intensive implantation approach to ensure that the electrodes covered the lesions to the maximum extent possible (Fig. 2). In addition, our coagulation contacts were within the resection range, which included the FCD lesion and the surrounding about 1 cm area. This meant that the coagulation area in our study included both the lesion and the perilesional area. This was similar to the concept of “extended lesionectomy” proposed by Wagner et al.^29^ Their extension commonly measured 5–10 mm around the MRI visible region. According to this range, 92% of patients achieved seizure cessation. In patients with FCD, perilesional discharges were considered a common phenomenon and could be recorded at a maximum distance of 4.8 mm.^30^ Although there was argument over whether perilesional area need to be treated, this slightly enlarged coagulation or resection had a good effect.

In our study, there was a correlation between MRI‐positive or ‐negative results and the RFTC outcome (the seizure‐free rate and response rate). Additionally, there was a correlation between the efficacy of RFTC and the sub‐categorization of etiology based on MRI findings. However, consistent with other literature reports,^28^, ^31^ we did not find any correlation between duration, age, side, and anatomic location of the lesion, and seizure frequency with treatment outcomes.

Bourdillon et al. found that procedure involvement of the occipital lobe was a predictive factor (OR = 6.824) for a response to RFTC.^32^ However, in our study, the seizure‐free rate and response rate in patients with occipital lobe epilepsy were the lowest among all brain lobes. MRI positivity is the only predictor for the seizure‐free rate (OR = 16.079) and the response rate (OR = 8.003) in our study. As previously discussed, the explanation for this is that MRI‐positive regions are targeted with more electrode coverage.

## Conclusions

SEEG‐RFTC is a safe procedure and yields favorable outcomes in numerous cases of focal DRE. Almost half of the patients achieve seizure freedom, and two‐thirds of them exhibit a positive response at the last follow‐up. Although the outcomes are not yet comparable to those achieved through conventional resection, a considerable number of patients could potentially benefit from this less invasive method. The MRI feature and etiology of epilepsy are correlated with the seizure‐free rate and response rate. And MRI positivity is the predictor for good RFTC outcome.

## Author Contributions

Author contributions to the study and manuscript preparation include the following. Conception and design: Qiangqiang Liu. Acquisition of data: Qiangqiang Liu and Yanqing Cai. Analysis and interpretation of data: Qiangqiang Liu, Ziyu Mao, and Wenzhen Chen. Drafting the article: All authors. Critically revising the article: All authors. Reviewed submitted version of manuscript: All authors. Approved the final version of the manuscript on behalf of all authors: Qiangqiang Liu and Dake He. Statistical analysis: Qiangqiang Liu, Bin Chen, and Wenzhen Chen. Administrative/technical/material support: Yong Lu and Chencheng Zhang. Study supervision: Jiwen Xu and Dake He.

## Funding Information

This work is supported by the Shanghai Municipal Health Commission (Grant No. 202340291) and Shanghai Jiao Tong University Fund for Interdisciplinary Research for Medical Applications (Grant No. YG2021QN30), and Guangci Innovation Plan of Shanghai Ruijin Hospital (Grant No. GCQH2023073).

## Conflict of Interest

The authors declare that the research was conducted in the absence of any commercial or financial relationships that could be construed as a potential conflict of interest.

## Data Availability

The data that support the findings of this study are available from the corresponding author upon reasonable request.

## References

[1] Fiest KM , Sauro KM , Wiebe S , et al. Prevalence and incidence of epilepsy: a systematic review and meta‐analysis of international studies. Neurology. 2017;88(3):296‐303. doi:10.1212/WNL.0000000000003509 27986877 PMC5272794

[2] Asadi‐Pooya AA , Brigo F , Lattanzi S , Blumcke I . Adult epilepsy. Lancet. 2023;402(10399):412‐424. doi:10.1016/S0140-6736(23)01048-6 37459868

[3] West S , Nevitt SJ , Cotton J , et al. Surgery for epilepsy. Cochrane Database Syst Rev. 2019;6(6):CD010541. doi:10.1002/14651858.CD010541.pub3 31237346 PMC6591702

[4] Chassoux F , Mellerio C , Laurent A , Landre E , Turak B , Devaux B . Benefits and risks of epilepsy surgery in patients with focal cortical dysplasia type 2 in the central region. Neurology. 2022;99(1):e11‐e22. doi:10.1212/WNL.0000000000200345 35418453

[5] Talairach J , Bancaud J , Bonis A , Szikla G , Tournoux P . Functional stereotaxic exploration of epilepsy. Stereotact Funct Neurosurg. 1962;22(3–5):328‐331. doi:10.1159/000104378 13984743

[6] Guénot M , Isnard J , Ryvlin P , Fischer C , Mauguière F , Sindou M . SEEG‐guided RF thermocoagulation of epileptic foci: feasibility, safety, and preliminary results. Epilepsia. 2004;45(11):1368‐1374. doi:10.1111/j.0013-9580.2004.17704.x 15509237

[7] Li H , Zhang M , Lin Z , et al. Utility of hybrid PET/MRI in stereoelectroencephalography guided radiofrequency thermocoagulation in MRI negative epilepsy patients. Front Neurosci. 2023;17:1163946. doi:10.3389/fnins.2023.1163946 37378015 PMC10291085

[8] Li Y , Gao J , Ye Z , Mu J . Magnetic resonance‐guided laser interstitial thermal therapy vs. stereoelectroencephalography‐guided radiofrequency thermocoagulation in epilepsy patients with focal cortical dysplasia: a systematic review and meta‐analysis. Front Neurol. 2023;14:1241763. doi:10.3389/fneur.2023.1241763 37928136 PMC10625445

[9] Fan X , Shan Y , Lu C , et al. Optimized SEEG‐guided radiofrequency thermocoagulation for mesial temporal lobe epilepsy with hippocampal sclerosis. Seizure. 2019;71:304‐311. doi:10.1016/j.seizure.2019.08.011 31521052

[10] Fu KH , Wang YC , Lim SN , et al. Long‐term outcome of seizure control and neurologic performance after limited hippocampal radiofrequency thermocoagulation for mesial temporal lobe epilepsy. World Neurosurg. 2023;173:e18‐e26. doi:10.1016/j.wneu.2023.01.061 36693618

[11] Li K , Shi J , Wei P , He X , Shan Y , Zhao G . Stereo‐electroencephalography‐guided three‐dimensional radiofrequency thermocoagulation for mesial temporal lobe epilepsy with hippocampal sclerosis: a retrospective study with long‐term follow‐up. Epilepsia Open. 2023;9:918‐925. doi:10.1002/epi4.12866 PMC11145609 37968869

[12] Moles A , Guénot M , Rheims S , et al. SEEG‐guided radiofrequency coagulation (SEEG‐guided RF‐TC) versus anterior temporal lobectomy (ATL) in temporal lobe epilepsy. J Neurol. 2018;265(9):1998‐2004. doi:10.1007/s00415-018-8958-9 29943202

[13] Dai Y , Zhang H , Fan X , Wei P , Shan Y , Zhao G . Optimized SEEG‐guided three‐dimensional radiofrequency thermocoagulation for insular epilepsy. Acta Neurochir. 2023;165(1):249‐258. doi:10.1007/s00701-022-05401-9 36342542

[14] Mullatti N , Landre E , Mellerio C , et al. Stereotactic thermocoagulation for insular epilepsy: lessons from successes and failures. Epilepsia. 2019;60(8):1565‐1579. doi:10.1111/epi.16092 31206643

[15] Mirandola L , Mai RF , Francione S , et al. Stereo‐EEG: diagnostic and therapeutic tool for periventricular nodular heterotopia epilepsies. Epilepsia. 2017;58(11):1962‐1971. doi:10.1111/epi.13895 28880999

[16] Filipescu C , Landré E , Gavaret M , Zanello M , Pallud J . Bilateral periventricular nodular heterotopia: can seeg—guided radiofrequency thermocoagulations cure the epilepsy? Epileptic Disord. 2024;26(1):158‐160. doi:10.1002/epd2.20171 37877673

[17] Liu C , Zheng Z , Shao XQ , et al. Stereoelectroencephalography‐guided radiofrequency thermocoagulation for hypothalamic hamartoma: electroclinical patterns and the relationship with surgical prognosis. Epilepsy Behav. 2021;118:107957. doi:10.1016/j.yebeh.2021.107957 33872942

[18] Wang S , Zhao M , Li T , et al. Stereotactic radiofrequency thermocoagulation and resective surgery for patients with hypothalamic hamartoma. J Neurosurg. 2020;134:1019‐1026. doi:10.3171/2020.2.JNS193423 32302977

[19] Liu Q , Wang J , Wang C , et al. FreeSurfer and 3D slicer‐assisted SEEG implantation for drug‐resistant epilepsy. Front Neurorobot. 2022;16:848746. doi:10.3389/fnbot.2022.848746 35295674 PMC8918516

[20] Liu Q , Mao Z , Wang J , et al. The accuracy of a novel self‐tapping bone fiducial marker for frameless robot‐assisted stereo‐electro‐encephalography implantation and registration techniques. Int J Med Robot. 2023;19(2):e2479. doi:10.1002/rcs.2479 36346988

[21] Narizzano M , Arnulfo G , Ricci S , et al. SEEG assistant: a 3D slicer extension to support epilepsy surgery. BMC Bioinform. 2017;18(1):124. doi:10.1186/s12859-017-1545-8 PMC5324222 28231759

[22] Xu Y , Wang H , Zhao Y , Feng X , Wu L , Lou L . Stereoelectroencephalography‐guided radiofrequency thermocoagulation of epileptic foci in the eloquent motor cortex: feasibility, safety, and efficacy. World Neurosurg. 2022;164:e492‐e500. doi:10.1016/j.wneu.2022.04.133 35537694

[23] Wang D , Wei P , Shan Y , Ren L , Wang Y , Zhao G . Optimized stereoelectroencephalography‐guided radiofrequency thermocoagulation in the treatment of patients with focal epilepsy. Ann Transl Med. 2020;8(1):15. doi:10.21037/atm.2019.10.112 32055606 PMC6995730

[24] de Oliveira LP , Pérez‐Enríquez C , Barguilla A , et al. Stereo‐electroencephalography‐guided radiofrequency thermocoagulation in patients with MRI‐negative focal epilepsy. J Neurosurg. 2023;138(3):837‐846. doi:10.3171/2022.6.JNS22733 35962969

[25] Kikuchi K , Togao O , Yamashita K , et al. Diagnostic accuracy for the epileptogenic zone detection in focal epilepsy could be higher in FDG‐PET/MRI than in FDG‐PET/CT. Eur Radiol. 2021;31(5):2915‐2922. doi:10.1007/s00330-020-07389-1 33063184 PMC8043950

[26] Severino M , Geraldo AF , Utz N , et al. Definitions and classification of malformations of cortical development: practical guidelines. Brain. 2020;143(10):2874‐2894. doi:10.1093/brain/awaa174 32779696 PMC7586092

[27] Guo Q , Tan HP , Chen J , et al. Efficacy and safety of conformal thermocoagulation guided by stereotactic electroencephalogram in the treatment of epilepsy caused by focal cortical dysplasia in eloquent cortex. Zhonghua Yi Xue Za Zhi. 2021;101(41):3393‐3398. doi:10.3760/cma.j.cn112137-20210418-00927 34758542

[28] Bourdillon P , Cucherat M , Isnard J , et al. Stereo‐electroencephalography‐guided radiofrequency thermocoagulation in patients with focal epilepsy: a systematic review and meta‐analysis. Epilepsia. 2018;59(12):2296‐2304. doi:10.1111/epi.14584 30345535

[29] Wagner J , Urbach H , Niehusmann P , Von Lehe M , Elger CE , Wellmer J . Focal cortical dysplasia type IIb: completeness of cortical, not subcortical, resection is necessary for seizure freedom. Epilepsia. 2011;52(8):1418‐1424. doi:10.1111/j.1528-1167.2011.03158.x 21740420

[30] Wellmer J . Lesion focused radiofrequency thermocoagulation of bottom‐of‐sulcus focal cortical dysplasia type IIb: conceptional considerations with regard to the epileptogenic zone. Epilepsy Res. 2018;142:143‐148. doi:10.1016/j.eplepsyres.2018.02.009 29550061

[31] Cossu M , Fuschillo D , Casaceli G , et al. Stereoelectroencephalography‐guided radiofrequency thermocoagulation in the epileptogenic zone: a retrospective study on 89 cases. J Neurosurg. 2015;123(6):1358‐1367. doi:10.3171/2014.12.JNS141968 26090841

[32] Bourdillon P , Isnard J , Catenoix H , et al. Stereo electroencephalography‐guided radiofrequency thermocoagulation (SEEG‐guided RF‐TC) in drug‐resistant focal epilepsy: results from a 10‐year experience. Epilepsia. 2017;58(1):85‐93. doi:10.1111/epi.13616 27859033

